# Supplementary figures and images for: Modulation of PRC1 Promotes Anticancer Effects in Pancreatic Cancer
Source: Cancers (Basel). 2024 Sep 27;16(19):3310. doi: 10.3390/cancers16193310 (PMC11475828; doi:10.3390/cancers16193310)

**Figure 6A**

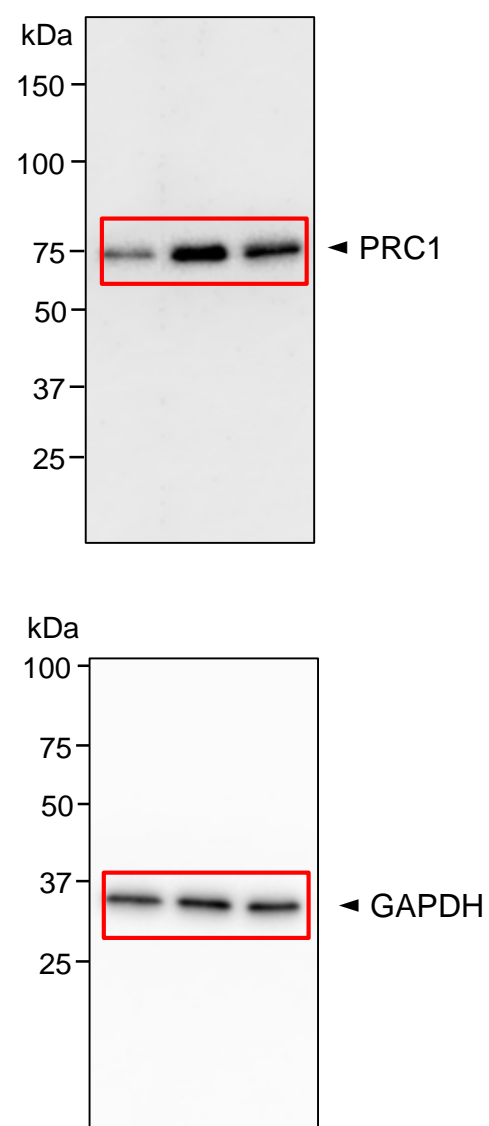

**Figure 6B (3 trials)**

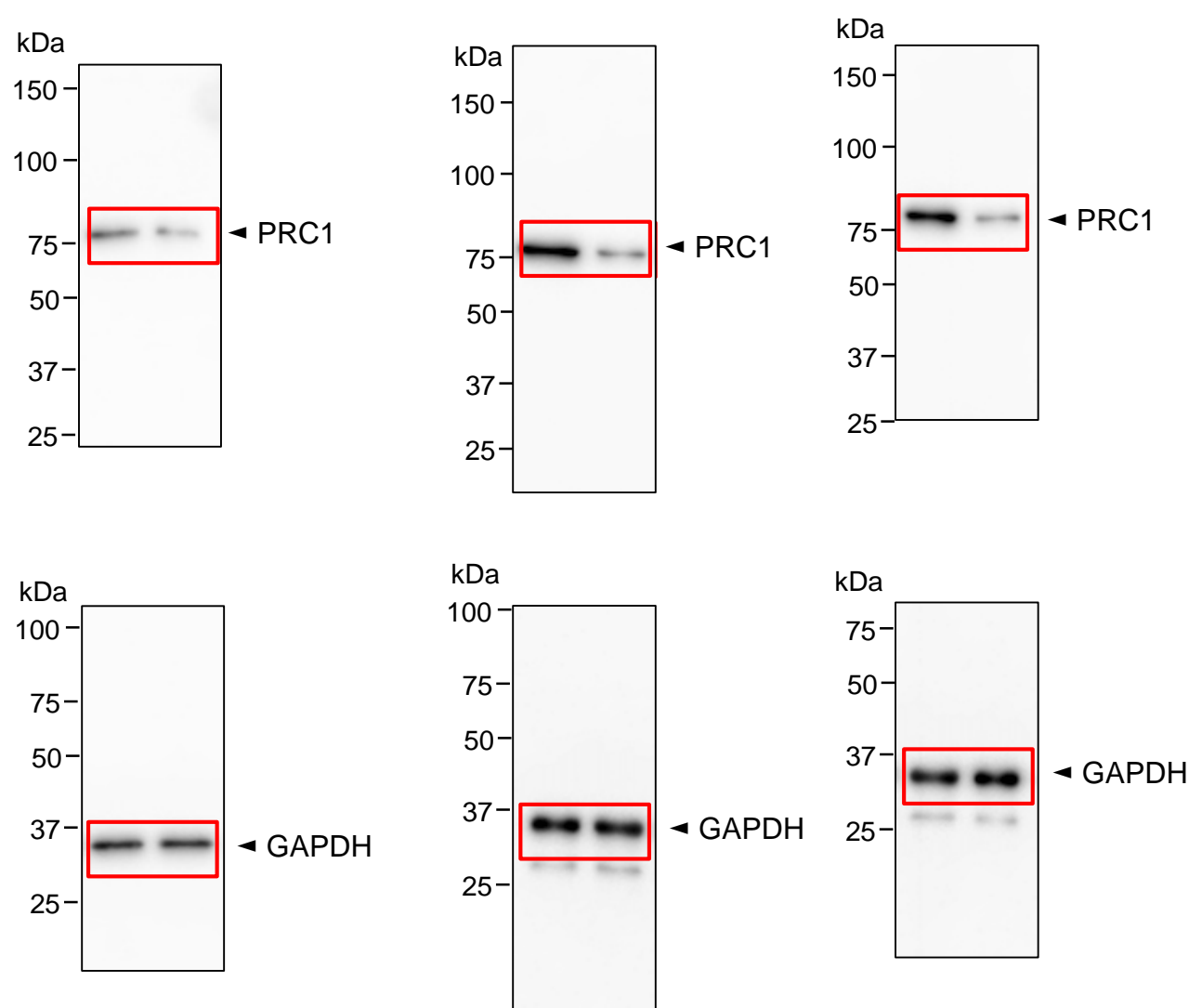

**Figure 6C (3 trials)**

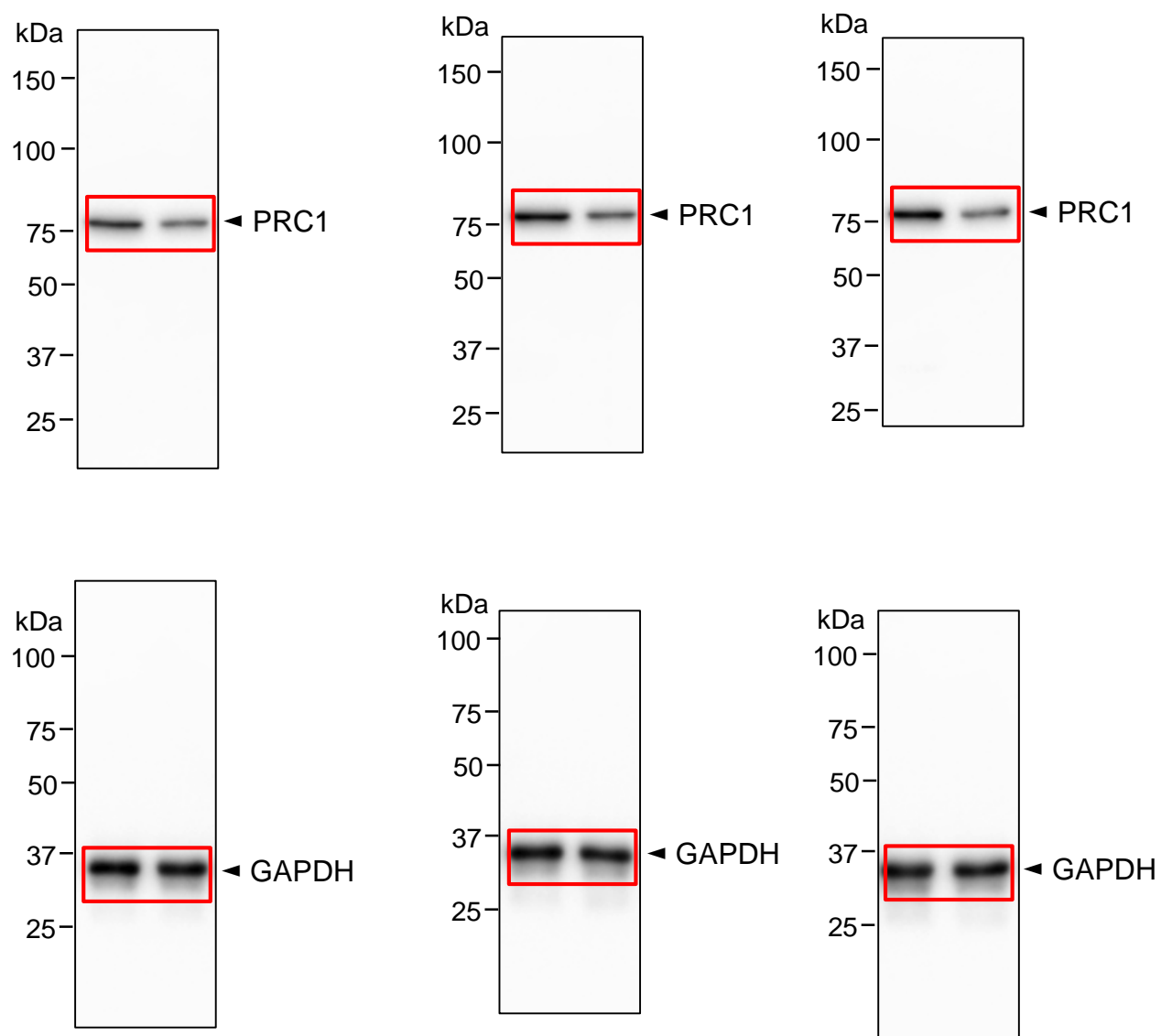

Supplement: Supplementary file 1 [file cancers-16-03310-s001.zip › cancers-3168477-supplementary.pdf]
